# Supplementary figures and images for: OLA1 is responsible for normal spindle assembly and SAC activation in mouse oocytes
Source: PeerJ. 2020 Jan 3;8:e8180. doi: 10.7717/peerj.8180 (PMC6944127; doi:10.7717/peerj.8180)

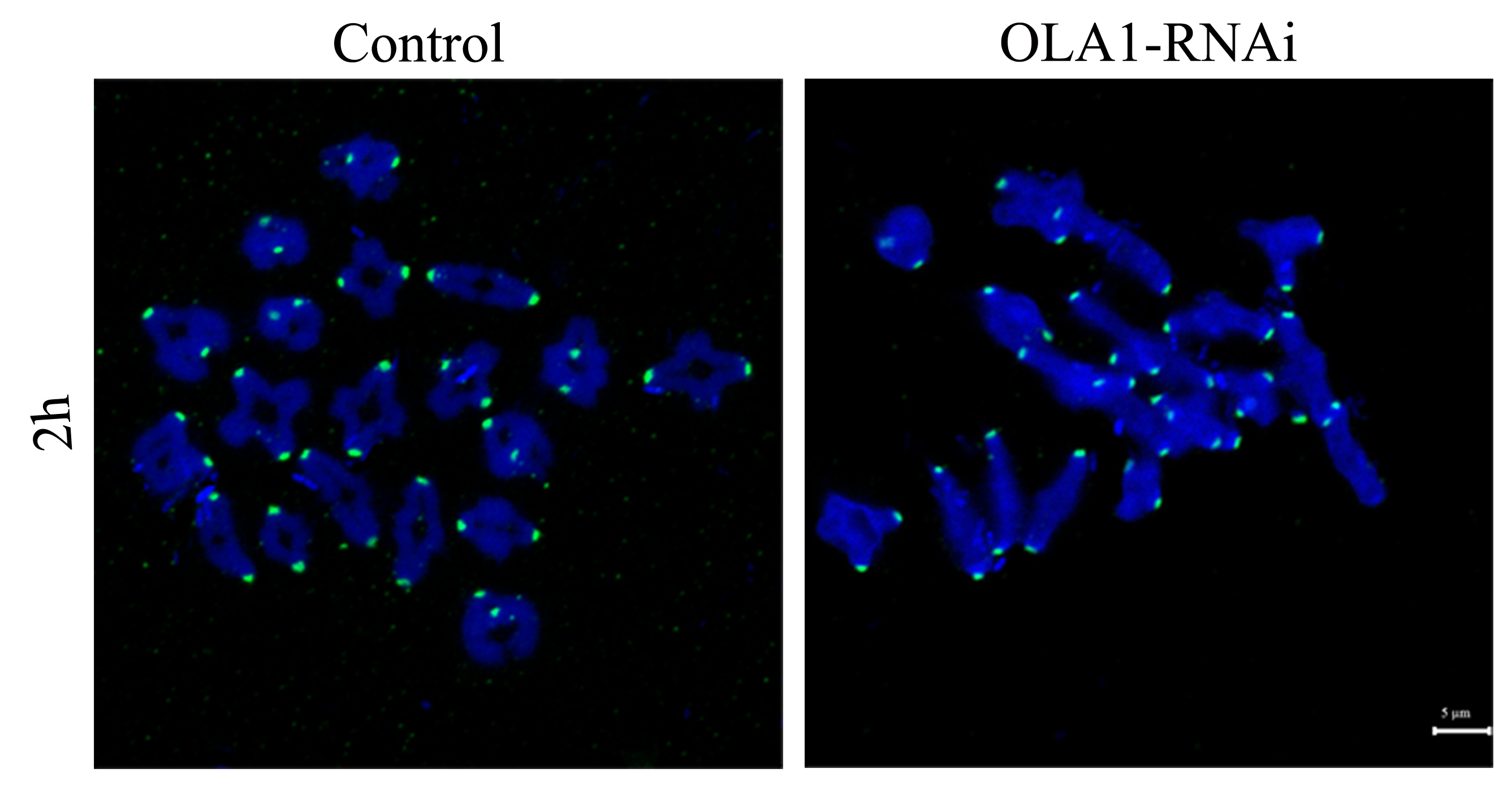

Supplement: File S2 — After knockdown, GV oocytes in control and OLA1-knockdown groups were released and cultured in pre-warmed M16 medium for 2 h, normally corresponding to GVBD stage. Then oocytes were collected for chromosome spreading and stained with BubR1. BubR1, green; Chromosome, Blue. Scale bar, 5 μm. [file peerj-08-8180-s002.jpg]

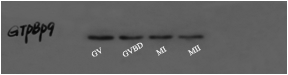

Supplement: Figure S1 [file peerj-08-8180-s003.tif]

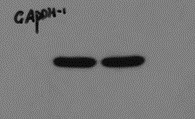

Supplement: Figure S2 [file peerj-08-8180-s004.zip › figure 2A/GAPDH.jpg]

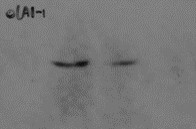

Supplement: Figure S2 [file peerj-08-8180-s004.zip › figure 2A/OLA1.jpg]
