# Supplementary material for: OLA1 is responsible for normal spindle assembly and SAC activation in mouse oocytes
Source: PeerJ. 2020 Jan 3;8:e8180. doi: 10.7717/peerj.8180 (PMC6944127; doi:10.7717/peerj.8180)
Supplement: Figure S2 [file peerj-08-8180-s004.zip › figure 2A/comment.docx]

We are regret that we are unable to provide the full-scan blots of Figure1B because these blots were original cropped when we transfer the gel to the PVDF membrane.
